# Supplementary material for: A progressive ratio task with costly resets reveals adaptive effort-delay tradeoffs
Source: bioRxiv. 2025 Jun 6:2025.06.03.657635. Preprint. [Version 1] doi: 10.1101/2025.06.03.657635 (PMC12157455; doi:10.1101/2025.06.03.657635)
Supplement: 1 [file NIHPP2025.06.03.657635v1-supplement-1.pdf]

bioRxiv preprint doi: https://doi.org/10.1101/2025.06.03.657635; this version posted June 6, 2025. The copyright holder for this preprint (which was not certified by peer review) is the author/funder, who has granted bioRxiv a license to display the preprint in perpetuity. It is made available under aCC-BY 4.0 International license.

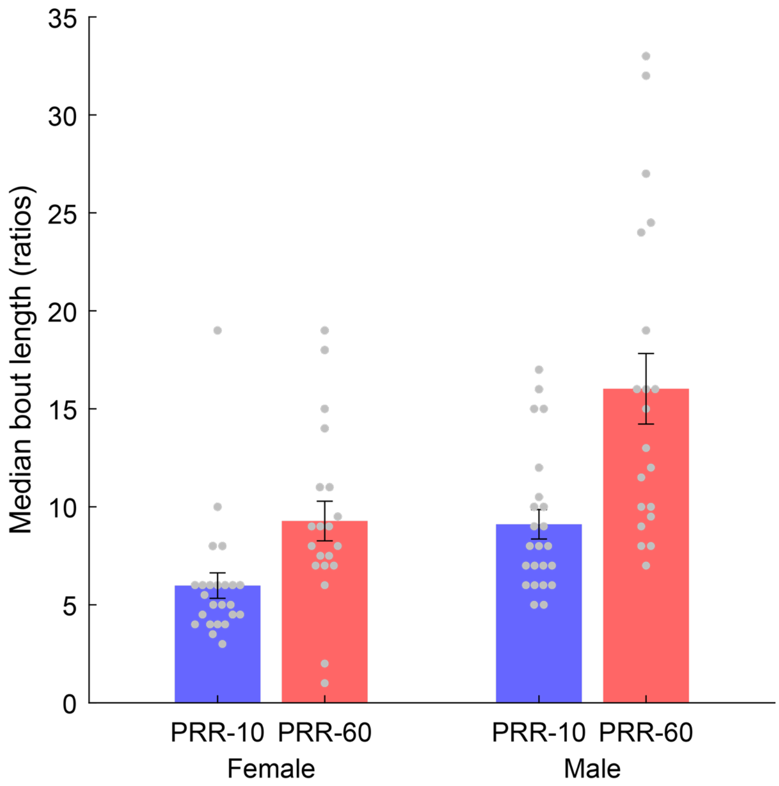

Figure S1. Reset delay affected bout length differently in male and female rats. Median bout length for PRR sessions is plotted separately for male and female rats. Each point indicates the median bout length in one session, and error bars indicated the mean ± SEM. General patterns are similar across male and female rats with longer reset delays eliciting longer bouts of work. However, this effect was strong in male rats, leading to a significant interaction between sex and reset delay (**Table 8**).
